# Bilateral hypoplasia of the long head of the biceps brachii muscle

**DOI:** 10.4103/0973-6042.80468

**Published:** 2011

**Authors:** Georgi P Georgiev, Lazar Jelev

**Affiliations:** University Hospital of Orthopaedics, “Prof. B. Boychev” Sofia, Bulgaria; 1 Department of Anatomy, Histology and Embryology, Medical University Sofia, Bulgaria

Sir,

Biceps brachii muscle (BB) is one of the most variable muscles in the human body in terms of the number and morphology of its heads.[1] In contrast, variations of the long or short heads of this muscle, including absence or variations in their insertions, are quite rare and could create difficulties in diagnosis at both magnetic resonance imaging (MRI) and surgery.[1–4]

**Figure 1:**
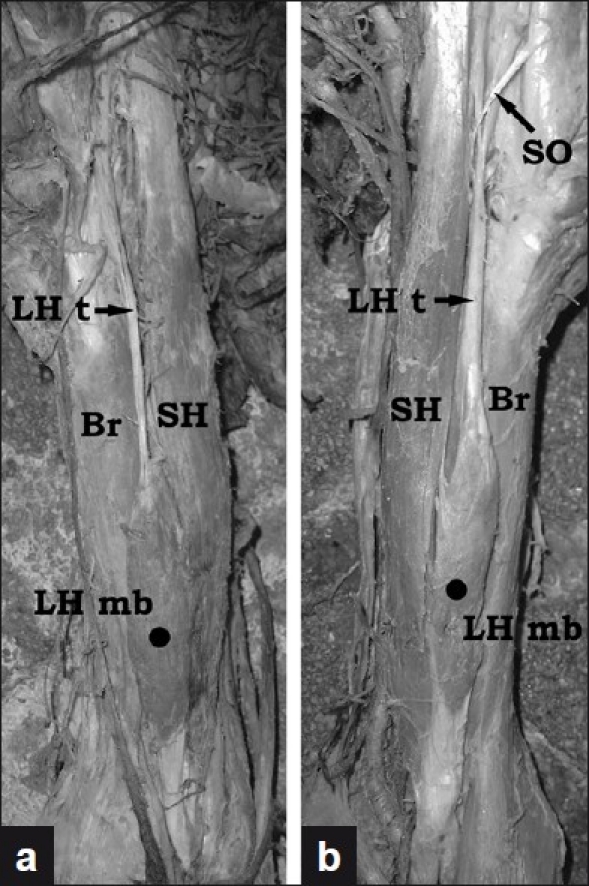
Photographs of the variant findings described on the right (a) and on the left (b) side muscles. (Br – Brachialis; LH mb – Muscular belly of the long head of the biceps brachii; LH t – Proximal tendon of the long head of the biceps brachii; SH – Short head of the biceps brachii; SO – Supernumerary origin of the short head of the biceps brachii)

In this report, we present a rare case of bilateral hypoplasia of the long head of the BB found during the anatomical dissection of a 58-year-old female cadaver. In both arms, the most remarkable findings were the strange-looking long heads [Figure 1 a–b], composed of abnormally long proximal tendons and small muscular bellies in the lower third of the brachium. The tendons had nearly equal lengths (right 12.2 cm, left 12.4 cm) but different widths – the left tendon (4.7 mm) was wider than the right one (3.4 mm). Proximally, both tendons were attached to the greater and lesser tuberosities of the humerus in the intertubercular sulcus (bicipital groove). Distally, the tendons continued in weak fusiform muscular bellies of similar size (length 11 cm, width 1.5 cm). In contrast, the short heads of BB on both sides were well developed. Each one arose as usual by a thick, flattened tendon from the apex of the coracoid process. Distally, the long and the short heads of both BB inserted to the radial tuberosities. Additionally, on the left side, the short head had a small supernumerary origin from the tendon of the pectoralis major [Figure 1b].

Anomalies of the long head of the BB include absence, duplication, deficiency and various origins (the capsular ligaments, the bicipital groove, the insertion of coracobrachialis, the tendon of the pectoralis major and the greater tuberosity of humerus).[1] Embryologically, the abnormal insertion could be explained with some interruption of the process of staged migration of the long head of the BB from a position between the fibrous capsule and the synovial layer.[2] Variations of the proximal segment of the long head of the BB are rarely encountered in clinical practice.[2] Flexion of the elbow with a variant long head, such as described here, may cause a swelling in the anterolateral aspect of the lower part of the brachium thus simulating a muscle rupture.[5] The unusual attachment of the long tendon of the BB, although rare, may also have a relationship with arthroscopic treatment of the superior labrum anteroposterior lesions.[4] The long head variations could be found during treatment of various shoulder disorders, including cuff degeneration, shoulder impingement and acromioclavicular joint arthritis.[2] Some authors speculate that different anomalies of the long head of BB may increase the risk of acquiring shoulder instability.[3,4] Variations of the long head may create diagnostic difficulty during shoulder arthroscopy[3] because the tendon is used as a landmark and may confuse even experienced surgeons.

The variations of the long head of the biceps tendon could also create diagnostic difficulty in MRI. However, awareness of their existence and MRI appearance can help prevent misdiagnosis, correct prospective MRI diagnosis and avoiding unnecessary surgery.[3]
